# A Serious Question for the Glasgow Infirmary Managers

**Published:** 1910-07-02

**Authors:** 


### The Hospital

A Journal of

The Medical Sciences and Hospital Administration.

New Series. No. 177, Vol. VII. [No. 1249, Vol. XLVIII.] SATURDAY, JULY 2, 1910

### A Serious Question for the Glasgow Infirmary Managers.

Not without reluctance do we recur to the action attributed to Sir William Macewen in forbidding the wearing of mourning bands by the nurses in his wards in the Glasgow Western Infirmary as part of the national mourning for the death of King Edward. We have, in our two previous issues, stated the facts, and these have not been contradicted, nor has any explanation been vouchsafed of a proceeding which everyone must allow to be exceptional and calculated to excite surprise. But for the brief telegram reprinted in our issue of June 25, Sir William remains silent, and any responsibility for the creation of what he calls a "false impression" rests therefore entirely with himself. It is true that certain writers in the Press have endeavoured to rush to his assistance, and have contributed from the realm of imagination various suggestions to account for his action. But he himself neither endorses nor denies these suggestions, and in so doing he leaves the field open to speculations which may come to have a quality not agreeable to himself. Beyond question he has taken a most unusual step, and one which could not fail to attract public notice; and yet to the public he apparently declines to afford any explanation. Our contention is that in so doing Sir William Macewen is failing to recognise his duty and the claim which may fairly and reasonably be made on him.

How do the facts stand? Sir William Macewen is an officer of an important public institution—namely, the Glasgow Western Infirmary. In accordance with general custom the Managers of this Institution arrange that the nurses shall wear an emblem of mourning, as other citizens have done, as an expression of the national grief for the death of a great and a good King. In no class of institution, one would think, could such an emblem be more seemly and appropriate than in the hospitals, in which King Edward took so practical an interest and to which he rendered services so valuable. All the other physicians and surgeons of the Western Infirmary recognised this, and the nurses in their several wards followed the wishes of the Hospital Managers. But in Sir William Macewen's wards this course, it is alleged, was forbidden. Yet he declines to explain what on the face of it is obviously an extraordinary proceeding. Now we contend that it is impossible to suffer this attitude on Sir William's part, and that the public has a right to know on what ground he, in a public institution, defied the wishes of the Managers and separated himself from his professional colleagues.

The matter, it must be remembered, is one in connection with which there is the strongest possible public feeling. The national mourning for the late King has had in conspicuous measure a unanimous quality and expression. And any individual who sets himself against this national current is manifestly doing something which cannot fail to produce a painful impression. Therefore the claim of the public in such a matter is strong and vital, and must be presented in insistent tones. No individual, however distinguished, can be permitted to act in opposition to the national sentiment and at the same time to refuse an explanation of his proceedings. What Sir William Macewen's explanation may be we do not pretend to know, but we do say that in refusing to produce it he is setting himself directly against the current of public opinion, and is assuming a right to which neither he, nor any other loyal subject, has any claim.

Some people have imagined, perhaps foolishly, that the action on which we are now commenting was due to some want of loyalty to the Throne, or that it may be regarded as indicative of failure to respect the memory of the late King. No one is suggesting, however, that it is necessary for Sir William Macewen to protest that he is a good and patriotic citizen. But he has taken action in his capacity as a public official in opposition to the general custom and sentiment, and therefore he owes an explanation to the public. Possibly he has already given such an explanation to the Managers of the Hospital in which he is one of the surgeons, or to the University authorities who are responsible for his nomination. It is conceivable that his position in relation to the King's household has demanded a similar step in still more exalted quarters. We venture to put it to him that his duty is not less to his fellow-citizens on whose generous sentiments his action has undoubtedly fallen with something like dismay. One of the Glasgow journals appears to think that the question here agitated is merely a fuss in a "nursing paper." Well, neither nurses nor editors ought to be ordered about at the crack of the whip, and both classes doubtless have their limitations and their ignorances. But we may take leave to say that we have some opportunity of estimating opinion in the great profession to which Sir William Macewen belongs, and we have yet to find a member of that profession who will defend his present silence. Our demand is framed in the public interest, and we know that it carries a large body of public support. We shall continue to press it until Sir William Macewen recognises that he cannot flout public opinion with impunity. If he has not already satisfied the Managers and the University for his conduct and indiscipline it rests with the authorities of these important bodies to demand and to publish an adequate explanation.

